# Exploring the intention to leave the nursing profession: a descriptive qualitative study in Italy

**DOI:** 10.3389/fpubh.2026.1833518

**Published:** 2026-04-30

**Authors:** Martina Saraga, Silvio Quirini, Angela Peghetti, Stefano Durante, Manuela De Rosa, Angela Vetromile, Lucia Golfieri, Andrea Turolla, Stefano Benini

**Affiliations:** 1 Translational Rehabilitation Sciences Group, Department of Biomedical and Neuromotor Sciences (DIBINEM), Alma Mater Studiorum University of Bologna, Bologna, Italy; 2 Professional Development and Implementation of Research in Health Professions Unit, IRCCS Azienda Ospedaliero-Universitaria di Bologna, Bologna, Italy; 3 Cardio-Thoracic and Vascular Anesthesia and Intensive Care Unit, IRCCS Azienda Ospedaliero-Universitaria di Bologna, Bologna, Italy; 4 Health Professions Direction, IRCCS Azienda Ospedaliero-Universitaria Bologna, Bologna, Italy; 5 AUSL di Bologna, Bologna, Italy

**Keywords:** high-intensity care, intention to leave, nursing, organizational wellbeing, qualitative research

## Abstract

**Introduction:**

Nurses and nursing assistants working in high-intensity hospital settings are exposed to demanding workloads, organizational pressure, and substantial emotional strain, factors commonly associated with job dissatisfaction and an increased intention to leave the profession. Intention to leave is a recognized predictor of actual turnover and is particularly relevant in high-intensity cardio-thoracic-vascular units, where care complexity and emotional burden are especially pronounced. This study aimed to explore the experiences, perceptions, and motivations of nurses and nursing assistants working in a high-intensity cardio-thoracic-vascular setting with regard to their intention to leave.

**Methods:**

A descriptive qualitative study was conducted at an Italian university hospital in accordance with the Consolidated Criteria for Reporting Qualitative Research. Four focus groups were carried out between May and June 2025 and involved nurses and nursing assistants employed in a high-intensity cardio-thoracic-vascular unit. All discussions were audio-recorded, transcribed verbatim, and analyzed using inductive thematic analysis. Methodological rigor was ensured through researcher triangulation and participant validation.

**Results:**

Five main themes emerged from the analysis: (1) motivation and professional identity, (2) working in high-intensity care, (3) interprofessional relationships, (4) symbolic representation of professional roles, and (5) intention to leave and job dissatisfaction. Participants described emotional exhaustion, workload imbalance, and a perceived lack of professional recognition as primary drivers of intention to leave. Conversely, strong teamwork, supportive peer relationships, and empathetic leadership were identified as protective factors.

**Discussion:**

Intention to leave among nurses and nursing assistants arises from the interaction of emotional, relational, and organizational dimensions. These findings highlight the need for interventions focused on supportive leadership, structured communication, and staff wellbeing initiatives to strengthen motivation, reduce attrition, and enhance the sustainability and quality of care in high-intensity clinical settings.

**Conclusion:**

These findings deepen understanding of intention to leave in high-intensity care and may inform future retention strategies.

## 1 Introduction

Nurse turnover, particularly intention to leave (ITL), represents a major challenge for healthcare systems worldwide and is especially critical in high-intensity clinical settings. Intention to leave refers to the predisposition of healthcare professionals to voluntarily leave their position as a result of organizational, personal, and emotional factors (1, 2). Extensive evidence indicates that ITL is one of the strongest predictors of actual turnover and is closely associated with adverse outcomes for healthcare organizations, including reduced continuity of care, increased workload for remaining staff, and compromised quality and safety of care (26).

In Italy, the nursing shortage has reached a critical level, reflecting a structural workforce deficit that threatens the sustainability of healthcare services across care settings. Despite recent efforts aimed at improving staffing ratios (ISTAT, 2022), Italy continues to report a number of practicing nurses per 1,000 inhabitants well below the Organisation for Economic Co-operation and Development average (3). This imbalance exacerbates work-related stress and intensifies turnover dynamics, particularly in high-intensity hospital environments.

High turnover is especially problematic in cardio-thoracic-vascular (CTV) units, where clinical complexity, technological intensity, and emotional burden are particularly pronounced. In these settings, staff turnover disrupts team cohesion and care continuity and generates additional organizational strain. This issue should also be interpreted in light of the organizational complexity of IRCCS settings, which are characterized by the coexistence of different healthcare professional profiles, with heterogeneous competencies and educational backgrounds (4). Excessive workload, lack of professional recognition, and emotional exhaustion have been consistently identified as key drivers of intention to leave (5). Moreover, high-intensity care environments amplify the risk of burnout and job dissatisfaction due to sustained exposure to critically ill patients, time pressure, and demanding organizational demands (2). Early career stages appear to be particularly vulnerable, with young nurses frequently reporting an early intention to leave the profession as a consequence of overwhelming stress, unmet expectations, and limited organizational support (6).

Understanding the determinants of intention to leave in high-intensity settings is therefore essential for the development of effective retention strategies. Evidence suggests that interventions targeting organizational support, leadership quality, and work–life balance play a central role in retaining skilled healthcare professionals and improving both staff wellbeing and care quality (7). However, much of the existing literature has focused on quantitative indicators, while fewer studies have explored the lived experiences and perceptions that shape professionals’ decisions to consider leaving, particularly within highly specialized units.

Against this background, it is important to consider the local organizational context. The cardio-thoracic-vascular department of the IRCCS University Hospital of Bologna is a high-intensity clinical setting that manages complex surgical and critical care pathways. Between 2021 and 2023, the department experienced marked fluctuations in its nursing workforce, with peaks of voluntary resignations concentrated in high-intensity units and operating theatres. These patterns highlighted a structural vulnerability to staff turnover and underscored the need for an in-depth exploration of the experiences and motivations underlying professionals’ intention to leave.

Based on these premises, the present study aimed to explore the motivations and experiences that lead nurses and nursing assistants working in a high-intensity cardio-thoracic-vascular unit of the IRCCS University Hospital of Bologna to consider leaving their job.

## 2 Methods

A total of 21 participants were included in the study, consisting of 18 nurses and 3 Nursing assistants (NAs) with purposive sampling continuing until data saturation was reached.

### 2.1 Aim

The aim of this study was to explore the experiences, perceptions, and motivations of nurses and NAs with regard to their ITL. The focus of the study was directed toward understanding how healthcare professionals experience daily work in a high-intensity care setting, with particular attention to the emotional, relational, and organizational factors that influence job dissatisfaction and the development of ITL. The research sought to capture both explicitly expressed accounts and underlying subjective meanings attributed to work experiences, in order to identify elements that may inform organizational strategies aimed at improving staff retention, professional wellbeing, and the sustainability of care in high-intensity clinical settings.

### 2.2 Study design

This study adopts a descriptive qualitative design. The research was conducted using focus groups to collect in-depth data on nurses’ and NAs’ work experiences within a high-intensity CTV unit at the IRCCS University Hospital of Bologna. This design was selected to allow an in-depth exploration of participants’ perceptions and lived experiences, providing a faithful and comprehensive description of the phenomenon under investigation without aiming to generate new theoretical models (8).

The study was promoted by Healthcare Management as part of a broader organizational improvement initiative, with the purpose of collecting data directly from healthcare professionals and supporting a bottom-up process grounded in everyday clinical practice within high-intensity care settings.

### 2.3 Theoretical framework

This study was informed by the Job Demand–Control model (Karasek, 1979) and the Job Characteristics Theory (9), which provided a conceptual framework to support the exploration of work-related experiences in high-intensity care settings. These theoretical models guided the development of the focus group discussion guide, helping to frame questions related to psychosocial demands, perceived control, motivational aspects, and work-related stressors associated with ITL among nurses and NAs working in the CTV unit.

The Job Demand–Control model emphasizes the interaction between high job demands and low decision latitude as a key determinant of work-related stress and burnout, while the Job Characteristics Theory highlights the role of task significance, autonomy, and feedback in shaping motivation and job satisfaction. Within the context of this study, these frameworks supported an in-depth exploration of participants’ experiences and perceptions without imposing predefined interpretative categories. Data analysis followed an inductive approach, allowing themes to emerge directly from participants’ narratives and ensuring fidelity to their subjective meanings and lived experiences.

### 2.4 Study setting and recruitment

The study was conducted in the cardio-thoracic-vascular (CTV) intensive care unit of the IRCCS University Hospital of Bologna, one of the leading clinical centers in Italy for the treatment of heart and lung diseases. Participants were recruited using purposive sampling, aimed at capturing a wide range of professional experiences among nurses and NAs working in this high-intensity clinical setting. Sampling decisions were guided by a balance table to ensure heterogeneity with respect to age, gender, years of service, and professional role, thereby reflecting the diversity of perspectives within the CTV unit.

An official institutional communication was disseminated through hospital channels to invite participation, intentionally over-recruiting to account for potential dropouts related to workload and shift patterns. A total of 47 professionals were initially invited to participate. Of these, 21 agreed to take part in the study (18 nurses and 3 NAs), resulting in a participation rate of 44.6%. Recruitment continued until data saturation was reached, defined as the point at which participants’ contributions became repetitive, dominant themes were consistently reinforced, and no new insights emerged from subsequent focus groups. This level of participation is consistent with qualitative studies conducted in complex healthcare settings, where organizational constraints and scheduling pressures often limit staff availability for focus groups (10).

### 2.5 Inclusion and exclusion criteria

The inclusion criteria were as follows: (a) registered nurses and NAs currently employed in the CTV unit with at least 6 months of work experience; and (b) ability and willingness to provide informed consent. Exclusion criteria included nurses or NAs in their probationary period or holding temporary contracts shorter than 6 months.

### 2.6 Data collection

Four focus groups were conducted, three with nurses and one with NAs, to foster open and horizontal discussion among participants. Each session was planned to include 8–10 nurses or 6–8 NAs and lasted approximately 2 h, in accordance with methodological recommendations for qualitative group facilitation (10).

The focus groups were moderated by an external hospital psychologist (L. G.), an experienced facilitator who had no direct working or personal relationship with the participants at the time of recruitment. Although some participants may have previously attended training sessions led by L. G. during their professional career, no close or supervisory relationship existed. A second observer (M. S.) supported the sessions by managing organizational aspects, taking field notes, and monitoring adherence to the discussion guide. Additional members of the research team contributed to the observation of group dynamics and the documentation of emergent content, thereby enhancing the credibility of the qualitative assessment.

Sociodemographic information was initially collected through an Excel table distributed via institutional email by the unit coordinator (A. V.) to all staff members of the operating unit. The table was completed individually and included age, gender, operational unit, years of service in the unit, professional role, educational qualifications, marital status, parental status, and geographical origin. Based on this dataset, purposive sampling was performed by L. G. and M. S. to ensure diversity and relevance of participants.

Before the focus group sessions, an informational meeting was held to explain the study objectives, procedures, voluntary participation, and withdrawal options, and to address participants’ questions. Written informed consent was obtained at this stage. Additional sociodemographic and professional information—including professional role, years of experience in the CTV unit, type of contract, and previous professional background—was collected through a brief structured form completed individually before entering the focus group room.

Each focus group followed a semi-structured discussion guide composed of open-ended questions designed to explore motivations, work experiences, and ITL. The guide was developed based on the theoretical framework and work psychology models in collaboration with the hospital clinical psychology team. No pilot focus group was conducted. Guiding questions invited participants, for example, to:

*describe their professional trajectory and motivations for working in a high-intensity CTV setting (e.g., “How long have you been working as a nurse or nursing assistant? How do you feel in your current role?”);*

*discuss factors contributing to turnover intention (e.g., “What are the main reasons that might lead a nurse or nursing assistant to consider leaving the profession or moving to another unit?”);*

*reflect on experiences of respect, recognition, and team dynamics (e.g., “How do relationships with colleagues and supervisors influence your job satisfaction?”);*

*explore the tension between professional passion and the desire to leave (e.g., “What factors make this job more or less sustainable and satisfying?”);*

*articulate symbolic representations of their current work experience (e.g., “Tell me the first word, image, color, or metaphor that comes to mind when you think about your work as a nurse or nursing assistant.”);*

*propose organisational improvements to enhance wellbeing and retention (e.g., “If you could change one aspect of your work environment, what would it be and why?”).*

All sessions were held in dedicated meeting rooms arranged with circular seating to promote interaction and a non-hierarchical climate. Focus groups were audio-recorded with participants’ prior written consent. Support materials, including observer grids, audio recorders, and consent and data processing forms, were prepared in advance. Consent forms were signed on the day of each session, and participants were informed of their right to withdraw up to the beginning of the discussion.

The four focus groups generated a total of approximately 6 h of audio recordings, corresponding to about 69 pages of verbatim transcripts, providing a rich and in-depth dataset for analysis.

### 2.7 Data analysis

In accordance with the qualitative nature of the study, no inferential statistical techniques were used for data analysis. Descriptive statistics were applied exclusively to summarize the sociodemographic characteristics of the sample, including age, gender, professional role, and years of experience. All focus group discussions were audio-recorded and transcribed verbatim, ensuring anonymity and confidentiality.

Data were analyzed using an inductive thematic analysis approach, as described by Braun and Clarke (11), allowing patterns and themes to emerge directly from participants’ narratives without the imposition of predefined analytical categories. The analytical process was conducted through several iterative phases:

a) *Familiarization with the data*, achieved through repeated listening to the audio recordings and in-depth reading of the transcripts to gain a comprehensive understanding of the content;
b) *Initial coding*, consisting of the identification of meaningful text units extracted from participants’ responses and organized in relation to the guiding questions;
c) *Labeling*, whereby each text unit was assigned a descriptive label summarizing its core meaning;
d) *Categorization*, through the grouping of labels with similar meanings into thematic categories representing shared cores of meaning;
e) *Theme development*, involving the refinement and synthesis of categories into broader themes capturing the key dimensions of the phenomenon under investigation;
f) *Validation*, carried out through independent review of categories and themes by two researchers (M. S., S. B.). In cases of disagreement, a third researcher (A. P.) was consulted to reach consensus and enhance methodological rigor.

To support analytic transparency, Table 1 illustrates the progressive abstraction process applied during thematic analysis, showing how raw data extracts were coded, grouped into sub-categories and categories, and ultimately synthesized into overarching themes. In addition, a thematic map was developed to summarize the main themes and visualize their interconnections. Selected quotations from participants were included in the Results section to preserve the richness and authenticity of their narratives.

**Table 1:** Exemplary coding tree.

Raw data extract	Label (code)	Sub category	Category	Theme
“…not long ago, I was at my station waiting for the doctors for the ward round […] they all gathered in the office […] we nurses were completely ignored […] I did not know what to do with my patient […] what am I for you? Just an executor?” (FG1, nurse)	Exclusion from clinical process and perceived devaluation of role	Professional identity and social utility	Professional dissatisfaction	*Intention to leave and job dissatisfaction*
“Sometimes I felt hurt […] you never stop learning, as I learn from the nurse I believe the nurse can also learn from me” (FG2, healthcare assistant)	Desire for mutual collaboration and interprofessional recognition	Relational dynamics and interprofessional collaboration	Interprofessional challenges	*Interprofessional Relationships and Team Dynamics*
“After 13 years of work and a master’s degree, I am exactly the same as 13 years ago” (FG3, nurse)	Professional stagnation	Lack of professional development	Professional dissatisfaction	*Intention to leave and job dissatisfaction*
“They transfer him and after 24 h you take him back for an emergency” (FG4, nurse)	Short-term readmissions	Managerial disorganization and ineffective planning	Work organization and shift management	*Working in a High-Intensity Environment*

Coding tree illustrating the process of thematic analysis from raw data extracts to labels, sub-categories, categories, and overarching themes.

Finally, the findings were compared with existing literature to contextualize the results within the broader scientific framework. Beyond its descriptive value, the analysis provided a practical foundation for the development of organizational improvement strategies aimed at enhancing the work environment, reducing turnover, and promoting the wellbeing of nurses and NAs working in high-intensity care settings.

### 2.8 Ethical considerations

Particular attention was paid to ethical aspects, in accordance with the principles of autonomy, confidentiality, and professional responsibility. All participants received detailed information about the study and provided written informed consent, ensuring voluntary participation, the right to withdraw at any time without consequences, the anonymity of statements, and the exclusive use of data for research purposes. Written consent was also obtained for audio recording and data processing prior to participation.

A separate consent form for the processing of personal data was prepared in compliance with the European Union General Data Protection Regulation (EU Regulation 2016/679, GDPR), guaranteeing confidentiality, secure data storage, and restricted access to authorized members of the research team only.

The study was conducted in full compliance with the ethical standards for research integrity and with the Professional Code of Ethics for Italian Nurses (FNOPI, 2025). The study protocol was reviewed and approved by the Bioethics Committee of the University of Bologna (Protocol No. 0076004, approved on March 11, 2025).

### 2.9 Rigor and reflexivity

Methodological rigor and data reliability were ensured through multiple strategies. With prior consent, all focus group discussions were audio-recorded and transcribed verbatim into Word documents. Transcripts were pseudo-anonymized using participant codes assigned at the time of enrollment. Once the accuracy of each transcription was verified, the corresponding audio recordings were permanently deleted.

The Word files containing transcripts, observation notes, and researchers’ memos were stored on a password-protected computer accessible only to the research team. Socio-demographic information was stored in separate files, distinct from the focus group data, to ensure confidentiality.

Throughout the analytical process, the researchers maintained reflexive diaries to document methodological decisions and reflections, supporting awareness of potential biases and enhancing analytical transparency. The research process adhered to the principles of transparency, consistency and credibility, ensuring fidelity to participants’ narratives. Although the focus groups were moderated by an external facilitator with no direct supervisory relationship with participants, reflexivity remained an essential component of the research process.

The researcher who attended the sessions as a non-participant observer and later contributed to data analysis consciously adopted a reflexive stance, seeking to bracket personal assumptions and minimize interpretive bias. Furthermore, repeated listening to the audio-recordings over time, after pseudonymization of participants, made it possible to revisit the material with greater analytical distance and reduced personal identification with individual speakers. This facilitated a more impartial interpretation focused on the meanings and patterns emerging from the narratives rather than on the participants as individuals.

All study-related materials will be securely retained for 5 years, as stated in the informed consent form. This study was conducted and reported in accordance with the Consolidated Criteria for Reporting Qualitative Research (COREQ) (12). These strategies contributed to the trustworthiness and methodological consistency of the study findings.

## 3 Results

### 3.1 Participant characteristics

The study included a total of 21 healthcare professionals working in the CTV Department of the IRCCS University Hospital of Bologna. The sample consisted of 18 nurses (86%) and 3 NAs (14%), all employed in high-intensity care units.

The overall mean age of participants was 40.1 years (range 25–63). Nurses had a mean age of 39.7 years (range 25–52), whereas NAs were older, with a mean age of 48.3 years (range 41–63). Most participants were female (66.7%), reflecting the gender distribution commonly observed in nursing and healthcare professions.

Participants reported a mean work experience of 10.9 years (range 1–34), ensuring representation of both early-career and long-tenured professionals. Regarding educational background, most nurses held a Bachelor’s degree in Nursing (*n =* 14), two had obtained a Master’s degree, and two senior nurses held the former professional nursing diploma. Among NAs, two had completed upper secondary education, while one had lower secondary education.

In terms of family status, 15 participants (71.4%) were single and 6 (28.6%) were married or cohabiting. Seven participants (33.3%) had children, for a total of 12 children.

Data collection resulted in four focus groups: three involving nurses and one involving NAs. Group composition was as follows: FG1 (5 nurses), FG2 (3 NAs), FG3 (6 nurses), and FG4 (7 nurses). The overall response rate was 44.6% (21 of 47 invited participants). Non-participation was mainly due to annual leave, illness, or incompatible work shifts.

Table 2 summarizes the sociodemographic and professional characteristics of the participants, including age, gender, years of service, and educational level, providing a transparent overview of the sample composition.

**Table 2:** Characteristics of participants.

Characteristics	Nurses (*n =* 18)	Healthcare assistants (*n =* 3)	Total (*n =* 21)
Mean age (years)	39.7 (25–52)	48.3 (41–63)	40.1 (25–63)
Gender	10 F (55.6%)/8 M (44.4%)	3 F (100%)	14 F (66.7%)/7 M (33.3%)
Mean years of service	11.4 (1–34)	9.9 (2–14)	10.9 (1–34)
Educational level	14 Bachelor’s; 2 Master’s; 2 Diploma	2 High school; 1 Lower secondary	–
Civil status	7 single; 7 married/cohabiting; 4 with children	1 single; 2 married; 3 with children	–

Values in parentheses represent minimum–maximum ranges.

### 3.2 Thematic findings

**Figure 1:**
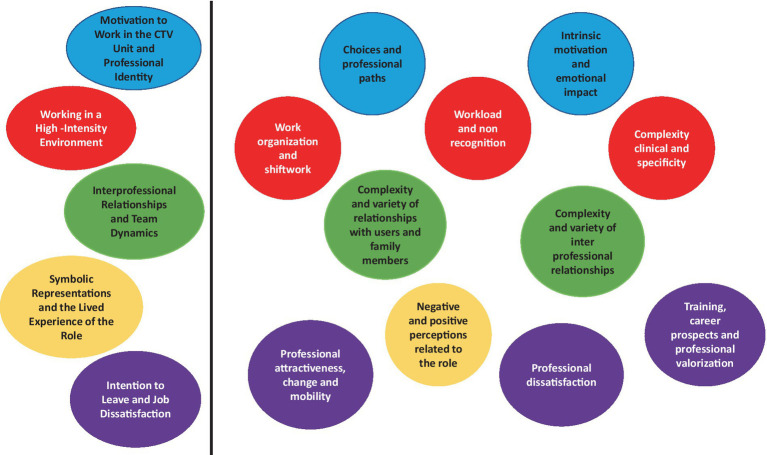
Illustrates the thematic map developed during analysis, summarizing the five main themes and their interconnections and highlighting the multidimensional nature of ITL, encompassing organizational, relational, emotional and symbolic dimensions.

An inductive thematic analysis of the four focus groups led to the identification of five main themes describing intention to leave (ITL) among healthcare professionals working in the high-intensity CTV area. The analysis followed a stepwise process from transcription and coding to categorization and thematic synthesis, until data saturation was achieved. The resulting thematic map reflects the multifactorial and interconnected nature of the phenomenon, encompassing personal, organizational, relational, and symbolic dimensions (Figure 1).

#### 3.2.1 Theme 1—motivation to work in the CTV unit and professional identity

The first theme concerns the motivations that led participants to choose the nursing or NA profession and, specifically, to work in a high-intensity CTV environment. Many participants described this choice as conscious and value-driven, associated with the desire to learn, acquire new skills, and contribute meaningfully to patient care.

*“I wanted to change, to learn new things and see more… I was lucky to have the chance to work in high-intensity care”* (NA, FG2).

A strong vocational and social dimension emerged, with several participants highlighting their commitment to helping others and contributing to the community:

*“I’ve always sought improvement both professionally and in helping people; that’s what motivates me”* (Nurse, FG1).

The intrinsic satisfaction derived from caring for others and witnessing patients’ recovery was described as a source of meaning and emotional reward:

*“It’s a job that gives back a lot, even just seeing the patient getting better”* (NA, FG2).

Although this value-driven approach was predominant, a smaller number of participants reported entering the profession primarily due to opportunity rather than vocation, developing a sense of belonging only over time. These accounts suggest that professional identity is not always an initial premise but may evolve through experience within the role.

Overall, professional identity appeared deeply rooted in ethical and emotional values, sustaining motivation while simultaneously exposing individuals to frustration when organizational conditions deteriorated.

#### 3.2.2 Theme 2—working in a high-intensity environment

This theme explores the organizational conditions characterizing daily work in high-intensity CTV settings, particularly shift management, rotation across units, workload, and their impact on personal life. Nurses reported rotating among three highly specialized areas—the Cardiac Surgical Intensive Care Unit, the Cardiology Unit, and the Pediatric Cardiac Surgery Unit—each requiring distinct competencies and levels of clinical acuity. Continuous alternation between these settings was described as a major source of stress.

*“I can’t work in the afternoon in the cardiology unit and the next morning in intensive care—it’s a completely different kind of job”* (Nurse, FG1).

Night shifts were perceived as particularly burdensome and associated with fatigue and sleep disruption:

*“Night shifts throw me off balance every time; it’s like taking an intercontinental flight”* (Nurse, FG1).

Participants also reported a progressive expansion of responsibilities without adequate training:

*“Every day we get new tasks and responsibilities, but no one trains us for them”* (Nurse, FG3).

Both nurses and NAs emphasized the increasing clinical complexity of patients over time, requiring constant vigilance and contributing to physical and mental exhaustion:

*“The more time passes, the more severe the cases become… you leave here really tired, without energy”* (NA, FG2).

A recurring narrative concerned the loss of care time, replaced by productivity pressures and speed-oriented workflows that limited relational care:

*“The priority is to be quick—the operating room is ready and you must send the patient down immediately”* (Nurse, FG3)

*“We’ve lost care time; now we’re always in a hurry, everything has to be done immediately”* (Nurse, FG3).

These organizational conditions also affected personal life, altering daily rhythms and the perception of time:

*“In there, you don’t even know if it’s raining or sunny… you lose track of time”* (NA, FG2).

While the overall experience was described as demanding and often unsustainable, some participants highlighted specific organizational elements—such as clearer shift planning, greater continuity within settings, or stable staffing—that temporarily alleviated workload. These nuances did not contradict the dominant narrative but illustrated variability within a generally critical context.

#### 3.2.3 Theme 3—interprofessional relationships and team dynamics

Interprofessional relationships strongly shaped daily experiences within the CTV units. Communication with families, particularly in pediatric settings, was described as emotionally demanding, especially when time and space were insufficient for adequate dialogue:

*“Parents stay all day, and we have no time or space to explain things properly”* (Nurse, FG1).

Several nurses recalled a past atmosphere of cohesion and mutual support that they perceived as progressively weakening:

*“We used to be like a big family; now we’re twenty in a shift, and I don’t even know 40% of their names”* (Nurse, FG4).

NAs often reported feeling undervalued, despite their essential contribution to daily ward functioning:

*“It seems like our work is always secondary, even though we provide everything needed to make the ward function”* (NA, FG2).

Relationships with physicians and management were frequently experienced as hierarchical, with decisions perceived as top-down and lacking staff involvement:

*“Decisions are made without listening to us—we’re left to manage what others have decided” (*Nurse, FG4).

Despite this prevailing sense of fragmentation, some participants described episodes of genuine collaboration with physicians or coordinators, indicating that positive relational dynamics existed but were inconsistently distributed across teams and shifts.

#### 3.2.4 Theme 4—symbolic representations and the lived experience of the role

The fourth theme explores the symbolic images used by nurses and NAs to describe their lived experience in high-intensity care. These metaphors conveyed emotionally charged representations of fatigue, disorientation, and identity strain, alongside occasional expressions of resilience.

Several participants used dark or destabilizing images to express emotional exhaustion:

*“I feel like a grey cloud”* (Nurse, FG1)

*“I feel like I’m in the middle of a desert”* (Nurse, FG1)

*“Quicksand—you sink and there’s no way out”* (Nurse, FG4).

Others described a sense of invisibility and impersonality, linked to diminished recognition:

*“There are so many of us that we don’t even know each other anymore… we seem like numbers”* (Nurse, FG4)

*“Our work often goes unseen”* (NA, FG2).

Alongside these negative representations, some positive and symbolic images emerged, reflecting enduring vocational meaning. One NA recalled being perceived by patients as:

*“an angel entering the room”* (NA, FG2), expressing gratitude and emotional connection. Another nurse described a sense of privilege:

*“I was lucky… this was the job of my life”* (Nurse, FG4).

Overall, these symbolic representations portray a role lived in tension between emotional strain and dedication, offering insight into how identity erosion may contribute to ITL.

#### 3.2.5 Theme 5—intention to leave and job dissatisfaction

The fifth theme integrates the previous dimensions into the outcome of ITL, described as a gradual and cumulative process shaped by organizational strain, declining motivation, and unmet expectations.

*“It’s not true that I never thought of leaving… I’ve considered going abroad”* (Nurse, FG1)

*“Every time someone resigns, I look at them with desire and admiration”* (Nurse, FG4).

Feelings of stagnation and lack of recognition were frequently reported:

*“After thirteen years and a master’s degree, I’m still in the same place—my only goal is to stop working nights”* (Nurse, FG3).

Participants also expressed dissatisfaction with training and career development opportunities:

*“Doing a refresher course after four years on a device you use every day feels insulting”* (Nurse, FG3).

A recurring element in participants’ narratives was the experience of organizational silence. Several reported avoiding expressing concerns or suggestions because they feared negative consequences or felt that their voice would not lead to any real change. This contributed to frustration, disengagement, and a gradual emotional withdrawal from the organization.

*“You say something and it comes back like a boomerang”* (Nurse, FG3).

*“In five years I have never seen a move toward improvement”* (Nurse, FG1).

Some participants also described experiences of moral distress, including situations in which they felt implicitly blamed for systemic problems:

*“Patients die because of you”* (Nurse, FG4).

At the same time, a few narratives highlighted the protective role of supportive colleagues and opportunities for learning, which partially mitigated the impact of stress and helped some professionals remain in the unit despite the difficulties.

Economic dissatisfaction and the perceived decline in professional status further contributed to ITL:

*“We’re probably the most underpaid in Europe”* (Nurse, FG4).

Ultimately, ITL emerged as the outcome of progressive disillusionment, in which moral commitment and professional passion were eroded by systemic inefficiencies and lack of recognition:

*“You start with vocation, but when that’s not enough anymore, leaving becomes the only way to breathe again”* (Nurse, FG4).

## 4 Discussion

This qualitative study explored nurses’ and NAs’ experiences and perceptions of intention to leave in a high-intensity cardio-thoracic-vascular setting. Findings show that organizational, relational, and emotional dimensions jointly contribute to the emergence of intention to leave, which appears as a gradual process of professional fatigue and disillusionment rather than as a single critical event.

These findings can be interpreted in light of the theoretical frameworks adopted in this study. In line with the Job Demand–Control model, participants described a work environment characterized by high job demands, including workload intensity, shift instability, and emotional strain, combined with limited decision latitude in relation to scheduling, organizational decisions, and everyday professional autonomy. This imbalance appears to contribute substantially to stress, dissatisfaction, and the progressive development of intention to leave (Karasek, 1979).

The findings are also consistent with Job Characteristics Theory. In particular, participants’ accounts pointed to reduced autonomy, limited feedback, and a diminished sense of task significance when care activities became fragmented, rushed, or insufficiently recognized. These conditions seem to undermine intrinsic motivation and job satisfaction, especially when professionals perceive a growing distance between the meaning traditionally associated with care and the actual experience of daily work in a high-intensity setting (9).

Participants described a progressive erosion of motivation in the face of hierarchical culture, limited communication, lack of recognition, and work–life imbalance: *“After thirteen years and a master’s degree, I’m still in the same place… my only goal is to stop working nights”* (Nurse, FG3). At the same time, a small number of professionals described themselves as *“lucky”* and still deeply attached to their role (*“This was the job of my life.”* – Nurse, FG4), representing divergent cases where vocation and meaning continue to counterbalance adverse conditions.

Organizational silence emerged as a key determinant, confirming the findings of Zou et al. (13), who interpret silence as a defensive strategy shaped by fear of consequences, resignation, lack of voice, and defensive adaptation. In our focus groups, several participants reported having *“stopped”* voicing criticism because it *“came back like a boomerang”* (Nurse, FG3) or because suggestions were perceived as systematically ignored: *“In five years I have never seen a move toward improvement”* (Nurse, FG1). Others highlighted the absence of genuine spaces for listening (*“It would be so useful just to share how we feel sometimes.”* – Nurse, FG4). For most, silence functioned as self-protection in a rigid, non-responsive environment; however, a few participants mentioned individual leaders who did listen and intervene, indicating that more open climates can exist locally even within an overall culture of silence.

Our data also reflect the four retention priorities described by Boone et al. (14)—professional support, education and development, financial and infrastructural incentives, and regulation. Participants emphasized the lack of structured interdisciplinary meetings and the experience of working “like in a factory… *everyone doing their own sector*” (Nurse, FG4), as well as a leadership perceived as distant: *“Decisions are made without listening to us; we are left to manage what others have decided”* (Nurse, FG4). Excessive bureaucracy and digital overload further reduced time for direct care (*“I couldn’t even look at the patient all morning because of documentation.”* – Nurse, FG3). At the same time, some operators described pockets of good teamwork and informal mutual support that helped them *“hold on,”* showing divergent experiences where team cohesion partially mitigates strain.

Consistently with Al Zamel et al. (15), intention to leave was strongly related to work–life imbalance, excessive workload, poor social image of the profession, and lack of career advancement. Participants reported struggling to reconcile shifts with family responsibilities and feeling chronically forced to *“put life second because you are always working”* (Nurse, FG1). They also described the physical and mental toll of understaffing: *“After 50, no one works here in direct care anymore… you feel like vomiting from fatigue”* (Nurse, FG1). Both nurses and NAs perceived limited recognition and visibility (*“Our work often goes unseen.”* – HCA, FG2; *“Doctors treat me as if certain knowledge does not belong to me.”* – Nurse, FG1), and the absence of real career pathways (*“You always stay there, while responsibilities keep increasing.”* – Nurse, FG3). By contrast, a minority explicitly rejected the idea of leaving, stressing the meaningfulness of clinical practice and the gratitude of patients as reasons to remain.

The experiences of newly hired nurses align with Holtz et al. (16), who, using Transitional Shock Theory, highlight the abrupt confrontation between academic preparation and clinical reality. Participants described acceleration of care pathways (*“Everything has to be done at the speed of light.”* – Nurse, FG4), shortened orientation, and premature responsibility, contributing to feelings of inadequacy and institutional mistrust (*“They tell you that you must be flexible, but is the organization flexible with us?”* – Nurse, FG4). Some reported being implicitly blamed for systemic problems (*“Patients die because of you”*), reinforcing moral distress. At the same time, a few accounts emphasized the protective role of supportive colleagues and more structured learning opportunities.

The findings are also consistent with the theoretical model of Pyhäjärvi and Söderberg (17), which interprets intention to leave as the outcome of psychological contract violation. Repeated discrepancies between expectations and reality—lack of recognition, inequitable workload distribution, top-down management, and the impossibility of providing relational care—gradually undermine trust and professional commitment. In our data, these micro-violations were described not only in relation to the organization but increasingly in relation to the profession as a whole in high-intensity settings.

Finally, the results confirm the themes summarized by Bahlman-van Ooijen et al. (18) and the dynamics described by Ciezar-Andersen and King-Shier (19). Participants depicted an environment of constant rushing, loss of “time for care”, and a sense of being “numbers” rather than recognized professionals, with presenteeism, skipped breaks, and chronic fatigue normalized as part of the job. This experience also emerged through the symbolic representations described in Theme 4, where metaphors such as “grey cloud,” “desert,” and “quicksand” expressed fatigue, disorientation, and identity strain, while more positive images suggested that vocational meaning could still persist despite adversity. Generational differences further amplified tensions, as younger staff questioned the ideal of the “always available nurse” and were more inclined to consider exit strategies. Overall, the convergence between our data and international literature supports the view that intention to leave is a systemic phenomenon arising at the intersection of organizational structures, team climate, and symbolic representations of care.

### 4.1 Strengths and limitations of the work

The main strength of this study lies in the use of a qualitative design, which enabled an in-depth exploration of nurses’ and NAs’ experiences and perceptions of intention to leave. Focus groups facilitated the emergence of shared meanings, emotions, and collective representations that are often overlooked by quantitative approaches. The focus on a high-intensity cardio-thoracic-vascular setting also allowed a detailed examination of a complex and still underexplored clinical context.

Methodological rigor was supported through adherence to the COREQ guidelines, independent coding by multiple researchers, and member checking, which enhanced the credibility and confirmability of the findings. Participants were given the opportunity to review and validate the main themes, helping ensure that the interpretations accurately reflected their experiences. The inclusion of professionals with different roles and levels of experience further increased the richness of the data.

Some limitations should also be acknowledged. The study was conducted within a single institutional context and in a highly specialized clinical unit, which may limit the transferability of the findings to settings with different organizational cultures, levels of care intensity, or patient complexity. In addition, group dynamics inherent to focus groups may have constrained the expression of dissenting or minority viewpoints, although several deviant cases did emerge during the discussions. Finally, data collection was limited to a specific timeframe, thus providing a snapshot of an evolving phenomenon.

### 4.2 Recommendations for future research

The findings of this study suggest several directions for future research. Further qualitative studies could explore more deeply how workload, leadership, communication, professional recognition, and emotional strain interact in shaping intention to leave among nurses and NAs working in high-intensity settings. These areas are consistent with previous literature showing that both organizational and relational factors play a central role in professionals’ retention and wellbeing (7, 14, 18). Comparative studies across different units or hospital contexts may also help clarify which findings are specific to highly specialized settings and which are shared more broadly across clinical environments.

In addition, future research could further examine deviant cases, namely professionals who remain motivated despite adverse working conditions, in order to better understand resilience and protective factors. Longitudinal and mixed-methods studies may also be useful to explore how intention to leave evolves over time and how it relates to job satisfaction, burnout, and retention (5, 17).

### 4.3 Implications for policy and practice

The findings of this study highlight the need for organizational strategies that address not only workload and scheduling issues, but also the relational and symbolic dimensions of professional experience. Participants’ narratives suggest that intention to leave is influenced by emotional exhaustion, limited recognition, reduced involvement in decision-making, and difficulties in communication within the work environment. These findings are consistent with previous studies showing the relevance of work environment and burnout in shaping nurses’ intention to leave (5) and with qualitative evidence emphasizing the role of organizational and relational factors in professionals’ retention and wellbeing (7).

From this perspective, policies and organizational practices aimed at improving retention in high-intensity settings should pay greater attention to supportive leadership, clearer communication processes, and work environments in which professionals feel heard, valued, and supported (25). The importance of communication and team climate also emerges in the literature on self-sacrificing nursing cultures and their impact on recruitment and retention (19). In addition, approaches such as participatory scheduling may be relevant where work–life balance and perceived fairness in shift management are major sources of dissatisfaction (20). Structured communication and teamwork programmes may also offer useful directions for organizational development, particularly in settings where fragmentation and poor interprofessional dialogue are experienced as sources of strain (21–23). Likewise, transition support for newly hired staff may be especially relevant in high-intensity environments, where adaptation difficulties and early disillusionment can contribute to turnover intention (24). Overall, greater attention to these dimensions may contribute not only to staff wellbeing, but also to the overall sustainability of care in highly demanding clinical settings (14).

### 4.4 Relevance to clinical practice

From a clinical perspective, the findings highlight the importance of fostering work environments that promote open communication, emotional support, and reflective practice. Creating structured opportunities for dialogue and shared reflection may help professionals process emotional burden and reinforce a sense of belonging within high-intensity teams. Involving nurses and healthcare assistants in decision-making processes enhances professional commitment, continuity of care, and perceived responsibility, while mentoring and peer-support mechanisms serve as protective factors against emotional exhaustion.

Promoting a culture that values self-care rather than self-sacrifice is essential to safeguarding both staff wellbeing and patient safety in high-intensity settings. Clinical practice benefits when psychological safety, teamwork, and professional recognition are actively supported, allowing care providers to maintain motivation and resilience over time. Ultimately, translating these principles into everyday clinical routines can help shift intention to leave toward intention to stay, strengthening professional identity and contributing to sustainable, high-quality patient care.

## 5 Conclusion

This study explored intention to leave among nurses and nursing assistants working in a high-intensity cardio-thoracic-vascular setting at the IRCCS University Hospital of Bologna. Through focus groups, it highlighted how work overload, unsustainable scheduling, emotional strain, limited professional recognition, and generational differences jointly shape professionals’ wellbeing and retention.

Consistent with international evidence, intention to leave emerged as a multidimensional and progressive phenomenon, rooted in organizational, cultural, and relational conditions rather than in isolated individual factors. The findings underscore the need for organizational redesign grounded in fair and participatory scheduling, inclusive leadership practices, staff wellbeing initiatives, and structured mentoring pathways that support professionals across different career stages (27).

By giving voice to frontline staff, this study contextualized intention to leave within a complex clinical environment and identified actionable strategies to strengthen motivation and retention. Future efforts should include the development and validation of specific tools to measure intention to leave, collaborative planning with healthcare leadership, and longitudinal evaluation of organizational and professional outcomes.

Ultimately, addressing intention to leave requires a systemic approach that integrates active listening, shared decision-making, and continuous evaluation. Such an approach is essential to build sustainable work environments capable of retaining skilled professionals and ensuring safe, high-quality care in high-intensity clinical settings.

## Data Availability

The original contributions presented in the study are included in the article/supplementary material, further inquiries can be directed to the corresponding author.
